# Three cases of jackfruit allergy in Canada: a case series

**DOI:** 10.1186/s13223-025-01006-w

**Published:** 2026-01-30

**Authors:** Faith Wierenga, Manstein Kan

**Affiliations:** 1 University of Alberta, Edmonton, AB Canada; 2 University of British Columbia, Vancouver, BC Canada

**Keywords:** Jackfruit, Allergy, Anaphylaxis

## Abstract

Jackfruit allergies have been rarely reported in the literature. In this case series, three case reports of jackfruit allergy in British Columbia, Canada are presented. All three patients also had a history of food pollen allergy syndrome to birch related foods. With increasing global trade and immigration, jackfruit will become more popular in non-endemic areas. As birch is a major pollen in North America, we could expect more reported jackfruit allergy in the future.

## Background

*Artocarpus heterophyllus* (jackfruit) is a fruit native to India, South East Asia and South America [1]. Few cases of jackfruit allergy have been described in the literature, and to our knowledge, none have previously been reported in Canada. Of the eight reported jackfruit allergy cases, three involved patients with concomitant latex allergy, four involved patients with allergic responses to birch pollen, and one case did not include allergy testing [2–8]. We present three cases of jackfruit allergy confirmed from history and skin testing in Canada. All our patients have a history of seasonal allergies and confirmed food pollen allergy syndrome to birch pollen related foods.

## Case 1

A 56-year-old female was referred to our outpatient allergy clinic for evaluation of an allergic reaction to jackfruit.

She had consumed approximately a quarter-sized piece of fresh jackfruit and, within 10 min, developed throat tightness along with facial and orpharyngeal edema. She initially presented to urgent care clinic which immediately sent her to the Emergency Department (ED) by ambulance. In the ambulance she received two doses of 0.5 mg IM epinephrine, 5 mg of nebulized epinephrine and IV fluids after documentation of hoarse voice, soft palate swelling to the point where uvula and tongue were not visible, and wheezing.

In the ED her blood pressure and vitals were stable, although she still had documented eye, soft palate and facial swelling. She received 20 mg IV famotidine, methylprednisolone, nebulized epinephrine and diphenhydramine 50 mg IV. She was monitored in the ED overnight and discharged with oral prednisone and antihistamine.

Upon review, she has a history of allergic rhinitis and food pollen allergy syndrome to the birch-related foods including raw apples and carrots, but no history of systemic anaphylaxis reactions. She reported ingesting no other fruits, foods or medication at the time of her reaction apart from the fresh jackfruit. She has a history of anaphylaxis reaction to nonsteroidal anti-inflammatory drugs (NSAIDs).

**Fig. 1:**
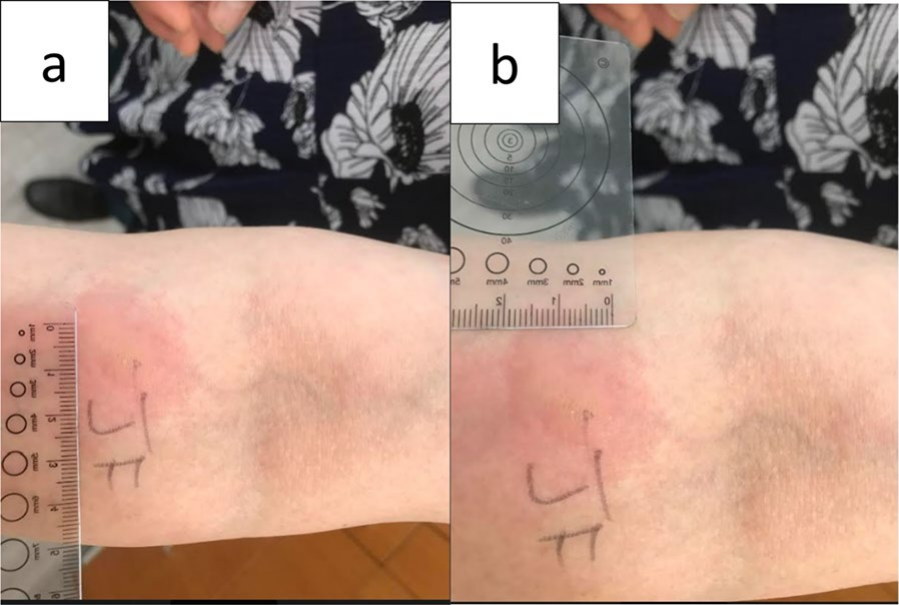
Patient’s positive response to jackfruit on epicutaneous testing for case 1

Epicutaneous skin testing to fresh jackfruit was positive [15 × 20 mm] (See Fig. 1a and b). Inhalant allergy testing was positive to alder and birch. Serum tryptase to assess for underlying mast cell disorder was negative. Histamine and 50% glycerinated human serum albumin [HSA]–saline were as positive and negative controls respectively.

The patient was advised to strictly avoid jackfruit. She was given an epinephrine autoinjector and an anaphylaxis action plan was reviewed with her.

## Case 2

A 15-year-old child developed throat tightness and difficulty breathing after consuming an estimated 10 by 10 cm sized piece of fresh jackfruit. He reported the symptoms as mild and did not seek medical attention. His symptoms resolved one hour after taking cetirizine.

Upon allergy review, he has not had jackfruit before the reaction and was born in Canada. He has seasonal allergies with positive skin testing to grass, alder and birch pollen. He experiences mild throat irritation to raw apples and cherries but no history of anaphylaxis. He has no history of asthma or other food allergies and tolerates latex.

**Fig. 2:**
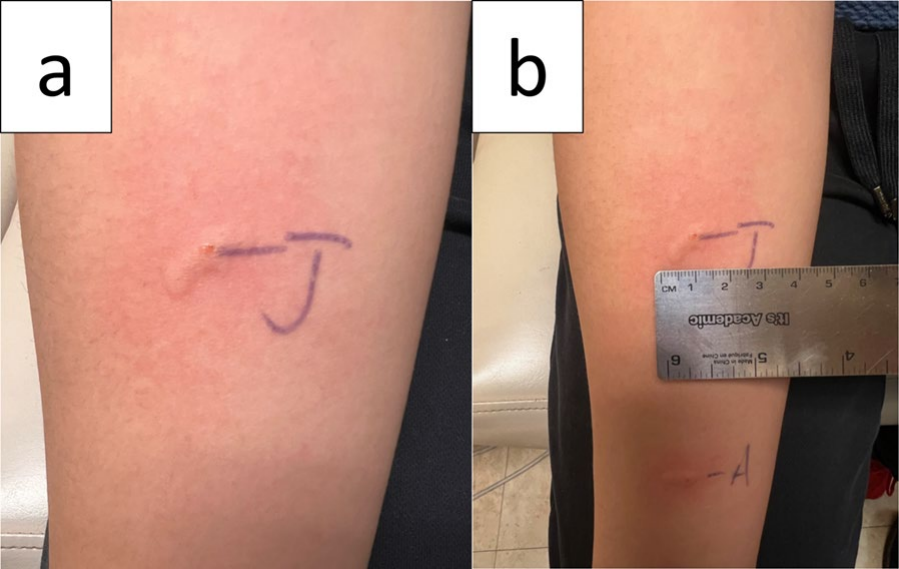
Epicutaneous skin testing results for case 2

Epicutaneous skin testing to fresh jackfruit was positive at 15 × 9 mm and fresh apple of 10 × 5 mm (See Fig. 2a and b). Other inhalant testing for him showed positive to alder, birch, grasses, dust mites and dog. Histamine and 50% glycerinated HSA–saline were used. The patient is advised to avoid jackfruit and was given an epinephrine auto injector to carry.

## Case 3

A 29-year-old reported a long-standing history of allergic reactions to jackfruit. She enjoyed the fruit as a child, but noticed since the age of 11 years, that she experienced an itchy throat and throat tightness with jackfruit. On many occasions the reactions resolved on their own as she was aware she should only consume small amounts of jackfruit at a time. In more severe instances she has resorted to taking antihistamines but did not require further treatment. She reported reactions to dried jackfruit chips as well as fresh jackfruit.

She has a history of asthma as well as seasonal allergies to grasses and birch pollen. She reported food pollen allergy syndrome reactions to birch-pollen related foods including cherries, apples and tree nuts (almonds, walnuts). Previous serum immunoglobulin E [IgE] to jackfruit was positive at 5.26 KU/L. Skin testing to dried jackfruit was negative. At the time of the writing the patient has not yet returned to the clinic for further testing.

## Discussion and conclusions

Our cases add to the increasing literature linking jackfruit as a birch pollen related fruit (Table 1). Bolhaar et al. reported two birch pollen sensitized patients with a severe allergic reaction to jackfruit [3]. Similar to our second case, neither of the patients reported consuming jackfruit before the reaction [3]. Immunoblot analysis to the two patients confirmed Bet V1 homology with jackfruit [3]. The study also recruited five birch pollen allergic patients without a history of reaction to jackfruit and found all of the patients skin tested positive to jackfruit. Jackfruit challenge to the patients also found they all developed food pollen allergy syndrome-like symptoms to jackfruit [3]. The study theorizes that a systemic response to jackfruit could be due to the stability of the PR10 protein in the digestive system, allowing it have a systemic absorption [3]. 

**Table 1:** Previously reported jackfruit allergy cases

Study	Location	Race and ethnicity	Participants	Allergy response	Positive test results	Positive birch
2	Switzerland	Filipino	1	Food pollen allergy syndrome	Birch Jackfruit Apple, peach, celery Alder, ash, hazel, grass mix, mugwort pollen	Yes
3	The Netherlands	Unknown	Patient 1: Patient 2:	Anaphylactic Food pollen allergy syndrome	Both: Birch Jackfruit Apple	Yes
4	USA	Jamaican	1	Anaphylactic	Birch Raw jackfruit Jackfruit peel	Yes
5	Thailand	Thai	1	Anaphylactic	Dried jackfruit Fresh jackfruit Latex glove brand #2 Latex glove brand #1	No
6	USA	White	1	Anaphylactic	Jackfruit Latex	No
7	USA	Bangladeshi	1	Anaphylactic	Jackfruit Latex	No
8	USA	Bangladeshi	1	Anaphylactic	Jackfruit	Not tested

Another large study tried to evaluate jackfruit homology to birch by skin testing 188 patients (with or without birch pollen allergy) to jackfruit [9]. They found 29 of 32 patients (91%) with a confirmed birch pollen allergy had positive skin testing to jackfruit [9]. The study was also able to identify Bet v 1 homology in Moraceae fruits by mass spectrometry [9]. More specifically, to jackfruit the homology in sequencing was at 76% to the Bet v 1 PR-10 proteins [9]. 

Multiple studies have reported a potential association of jackfruit allergy with latex. ^5,6,7^ Wongrakpanich et al. reported a 34-year-old nurse with anaphylaxis following the ingestion of dried jackfruit [5]. Skin testing was positive for jackfruit and latex gloves, along with other latex associated foods including kiwi and papaya [5]. Dey et al. also reported a 68-year-old woman who developed anaphylaxis to jackfruit [7]. She has a reported history of latex allergy with mild rash and also seasonal allergies [7]. Unfortunately no skin testing or serum IgE was done to confirm the jackfruit allergy or latex and birch sensitization. Hemmer et al. reported an association of hevein-like domains (HLD) with Moraceae fruits [9]. Of note, the study skin tested two patients with latex allergy with none reacting to jackfruit [9]. None of our reported patients have a reported latex reaction or allergy to its associated foods.

The identification of Bet V1 homology with jackfruit provides an immunological explanation for our three patients, all of whom are birch pollen sensitized and also react to other birch-related foods. It also provides an explanation to cases of severe jackfruit reaction without prior exposure [3, 9]. 

Several studies have attempted to immunoblot jackfruit allergen [3, 9, 10]. Most have identified an allergenic link to Bet v1 but it is possible a non-birch related allergenic protein mechanism may also exist [10]. This would explain the many cases of non-birch related jackfruit allergy [6, 7]. Further research into the area would be important to further advance our knowledge of jackfruit allergy.

We present three cases of jackfruit allergy associated with birch pollen related food allergy. With increasing global trade and immigration, jackfruit will become more popular in non-endemic areas. As birch is a major pollen in North America, we could expect more reported jackfruit allergy in the future.

## Data Availability

All data generated or analysed during this study are included in this published article.

## Abbreviations

ED: Emergency department
